# Strategies for Treating Compensatory Articulation in Patients with Cleft Palate

**Published:** 2014-03

**Authors:** Maria Del Carmen Pamplona, Antonio Ysunza, Santiago Morales

**Affiliations:** 1 Cleft Palate Clinic of the Hospital Gea González, Mexico City;; 2 Beaumont Health System, Royal Oak, MI, USA;; 3 Department of Psychology, Penn State University, USA

**Keywords:** Cleft palate, language, speech, therapy

## Abstract

Patients with cleft palate frequently show compensatory articulation (CA). CA requires a prolonged period of speech intervention. Some scaffolding strategies can be useful for correcting placement and manner of articulation in these cases. The purpose of this paper was to study whether the use of specific strategies of speech pathology can be more effective if applied according to the level of severity of CA. Ninety patients with CA were studied in two groups. One group was treated using strategies specific for their level of severity of articulation, whereas in the other group all strategies were used indistinctively. The degree of severity of CA was compared at the end of the speech intervention. After the speech therapy intervention, the group of patients in which the strategies were used selectively, showed a significantly greater decrease in the severity of CA, as compared with the patients in whom all the strategies were used indistinctively. An assessment of the severity of CA can be useful for selecting the strategies, which can be more effective for correcting the compensatory errors.

## INTRODUCTION

Several authors have described the speech disorders in patients with cleft palate (PCP). Some of these disorders are articulation impairments associated with the structural deviations in these patients (1), and are secondary to a velopharyngeal insufficiency (VPI).

These abnormal articulation patterns are usually referred as a compensatory articulation (CA). CA severely affects intelligibility and usually requires a prolonged period of speech pathology intervention (2, 3).

Speech disorders in patients with CP, such as CA, may initially occur as a consequence of the cleft. For this reason, many authors consider CA as a phonetic disorder (4, 5). However, CA can also be considered a phonologic disorder. It is described that over time, these errors can become incorporated into the child's developing rule system of articulation (6, 7).

Considering CA as a phonologic disorder has many implications. First, some researchers have indicated that the phonological system is integrated with the language system (8). It is suggested that the language of children with CA should also be assessed and treated during intervention. Second, for assessment, an analysis of phonologic processes in addition to phonetic analysis has to be included. Finally, for speech intervention, some scaffolding strategies have to be used during intervention. These strategies are aimed to modify the articulation system of each child.

The use of these strategies is useful for scaffolding the child’s communicative turns in order to increase his/her speech and language performance (8). Scaffolding strategies include various types of prompts, questions, information, restatements, and other procedures, which provide support to the child as he/she is actively engaged in the process of communicating a message (9).

Many authors have described scaffolding strategies for facilitating a better way to communicate and/or articulate the sounds of speech (7, 10). Only few of them have specified the expected time to apply each strategy in order to improve effectiveness. Modeling (8, 10), Phonemic Cues (9), Minimal Pairs (10, 12), Cycles (7), Imitation and drills (13), Requests for clarifications (14), Phonetic changes (9), think aloud in phonemic awareness (15) and Expansions (8), are some of the commonly used strategies.

However, when studying the efficacy of these strategies for treating CA in PCP, some result more adequate depending on the specific stage of speech development or severity of articulation impairment (15). Authors in that study concluded that strategies that include direct instruction such as phonetic change, cloze procedure with phonemic cues, and/or think aloud in phonemic awareness, appeared to be more effective for promoting positive changes in articulation in patients who were in the higher levels of severity of CA, such as those who can only articulate correctly isolated phonemes or used them in words or short phrases with the support of the clinician. When these strategies were compared with those that do not include direct instruction such as modeling or modeling with stress, a significant difference was observed. Moreover, these two strategies showed similar effectiveness in the lower levels of severity of articulation such as when children are able to articulate in familiar contexts, and even during spontaneous speech inconsistently. In such levels, children are more aware of the use of speech sounds, and show confidence for producing these sounds during a more structured discourse.

The purpose of this paper is to study and compare two different ways for using scaffolding strategies during intervention in PCP.

## MATERIALS AND METHODS

This study was carried out at the cleft palate clinic of the Hospital Gea González in Mexico City. The Bioethics and Research Committees of the Hospital approved the protocol and the study had been performed in accordance with the ethical standards laid down in the 1964 Declaration of Helsinki’s and its later amendments. Before the inclusion of each patient into the study group, the parents or legal guardians were carefully explained about the procedures and the methodology of the protocol. All parents of the patients included in the study group, agreed to participate in the study and gave their informed consent prior to the inclusion of the study.

Sample size was calculated at an Alfa of 95% confidence interval and a Beta power of 80% for a comparative study of two groups. The distribution of the severity of CA across the patients evaluated in our center during the last 2 years was considered.

The aim was to detect a difference of at least 20% between categories. According to these calculations, a minimum of 40 patients classified in each group should be included in the study.

All cleft palate patients attending the Speech Summer Camp 2013, organized by the Phoniatrics department of the cleft palate clinic of the Hospital Gea González in Mexico City were evaluated. To qualify for the study group, patients had to meet the following criteria:

- Unilateral, complete cleft of primary and secondary palate (UCLP) (16);
- No known neurological or genetic syndromes;
- No identified severe language disorders according to the SDS-Model evaluation practiced in our clinic routinely and reported previously (17);
- Palatal repair of the UCLP performed according to the surgical routine of the cleft palate clinic. This routine includes: surgical repair of the lip and primary palate between 1-3 months and surgical repair of the secondary palate between 4-8 months with a minimal incision palatopharyngoplasty (18);
- VPI after palatal repair demonstrated by clinical assessment, videonasopharyngoscopy and multi-view videofluoroscopy;
- CAD in association with VPI, had to be demonstrated during a complete phoniatric clinical evaluation;
- Absence of postoperative fistulae;
- Chronological age between 3 – 7 years of age at the time of selection for the study group;
- Normal hearing demonstrated by conventional pure-tone audiometry.

### Participants and procedures

All patients received a complete clinical evaluation of Speech, Language and Voice. It should be pointed out, that such evaluation is considered as the gold standard diagnostic indicator of compensatory articulation disorder.

A total of 90 patients were selected for the study. They were assessed at the onset and at the end of the 2011 speech summer camp, including an analysis based on the Whole Language Model and the basic Phonological principles. Especial attention was focused on the detection of compensatory articulation patterns, the placement and manner of articulation of these patterns and the phonological rules of the phonological system of each child. For this purpose, children were videotaped interacting with a trained speech pathologist during storytelling for 30 minutes. A 20 minutes segment was selected where a high level of verbal interaction occurred. The 20 minutes of interaction were transcribed verbatim for analyzing the presence and severity of compensatory articulation.

All speech & Language Pathologists (SLP) participating in this study had been performing phonological transcriptions of cleft palate children for at least 5 years.

For assessing the reliability of the evaluation of CA severity, as expressed by the scale used in our center, a blind procedure was utilized, whereby all analysis was independently conducted by two-trained SLP. Whenever there was a disagreement, each case was discussed until a consensus was reached.

The children were randomly divided into two groups. Patients assigned to the active group were matched by gender with patients included in the control group. The age range of the patients from both groups was kept as similar as possible. The level of articulation of all children was evaluated according with the clinical scale of severity of CA used in our center (19).

Both groups received speech therapy aimed to correct compensatory articulation according to the principles of the Whole Language Model (3).

Storybook readings were the main context for intervention. Intervention was aimed to reinforce correct speech sounds while enhancing cognitive linguistic organization. The treatment goals were set depending on the phonological rules that were active in the child’s system (12), and the intervention program was focused on the modification of groups of sounds that seemed to be treated by the child in a similar fashion. In other words, errors were attacked at the rule level, rather than at a phonetic level (e.g. all plosives substituted by glottal stops).

Within these main principles of intervention, in the first group (control group), the SLP used the following strategies described for phonologic intervention: modeling, modeling with stress, phonemic cues, cloze procedure, phonetic changes, and think aloud in phonemic awareness. The SLP was instructed that all strategies should be attempted in each case. In contrast, in the second group (experimental group) the SLP used the same phonologic strategies, but they were selected according with the level of severity of CA as assessed by the scale described herein. (15) (Table 1).

**Table 1:** Use of strategies in experimental group for correcting articulation (CA)

Level of articulation	Characteristics of the level	Strategies

Constant CA	The patient is not able to correct articulation not even in isolated phonemes and despite direct instruction.	Phonetic changes, Think aloud in phonemic awareness.
Articulation in isolated phonemes	The patient is able to correct articulation only in isolated phonemes through direct instruction.	Phonetic changes, Cloze procedure with phonemic cues, Think aloud in phonemic awareness.
Articulation with strategies	The patient can correct articulation during isolated words or selected short phrases, only when the clinician uses specific phonologic strategies.	Phonetic changes, Cloze procedure with phonemic cues, Think aloud in phonemic awareness, Modeling with stress.
Articulation within context	The patient self-corrects articulation when using speech within a specific context. For example during telling a story from a story book which the patient already knows well. Nonetheless he shows frequent compensatory errors during spontaneous speech, and this affect intelligibility.	Modeling with stress, Modeling.
Inconsistent articulation	The patient shows compensatory articulation errors inconsistently during spontaneous speech. Intelligibility is not significantly affected.	Modeling with stress, Modeling.
Appropriate articulation	The patient is able to produce adequate placement and manner of articulation during spontaneous speech, including non-present situations.	

The following variables from both groups were compared: age at the onset of speech therapy, level of articulation at the onset and at the end of the study, and advanced levels of articulation.

### Speech Summer Camp

The speech summer camp is carried out for a period of 4 weeks. PCP attended 4 hours daily. The objective was to offer intensive speech therapy. The methodology followed was “narrative-centered themes” (20). This procedure serves as a means for addressing children’s language and articulation in an integrated manner. Thus, articulation goals were always present in an organized whole. Most activities were realized as event representations such as storybook reading, art and/or cooking activities. In all activities, speech language pathologists used different strategies for working in articulation. These strategies serve to assist the child in formulating messages with greater complexity, specificity of meaning, accuracy, and clarity of expression (20).

Children were divided in small groups depending on age, linguistic development, and cognitive level. All strategies were used within structured activities to provide children with contextually appropriate opportunities to use language and focus on articulation. The clinician could choose either to expand an expression or refine upon an expressed idea by giving specific information.

Clinicians also used verbal expansions to provide children with information about higher levels of language organization. This type of interaction has been shown to increase the semantic and syntactic complexity of children’s utterances, and may have similar effects on articulation (8).

### Strategies for articulation

The following strategies were used during the summer camp:

**Modeling.** Modeling is one of the most frequently reported strategies. The speech pathologist models the speech and/or language behavior that the child is to learn. Some clinicians found that children respond spontaneously if the language behaviors being stressed are at the child’s level. Thus, they model the behavior but do not normally request or formal elicitation. Modeling approaches the normal conditions of language learning more closely than the elicited imitation procedure (11). Example: Child: “_all he_ (call her)”. Clinician: “Yes, call her”. This strategy has been often described as useful for language development, but it is also proposed as a strategy for children with articulation impairment. Hoffman states that the models of appropriate articulated words in context are thought to help stimulate appropriate syllabic and phonemic organization (8). The clinician has the opportunity to model and talk about the structure of words within an overall communicative context (21).

**Modeling with stress.** With this strategy, the speech pathologist models targeted sounds of speech, but includes a brief pause before the sound and also, a stress on the sounds (phonemes) the clinician wants to model. For example, during a play situation in which a cake is being cut, the child says “_ut the _ae_(cut the cake); The clinician can respond “yes, (pause)….cut the (pause)…cake. I will (pause)…cut the (pause)…cake and give you a (pause)…piece”, stressing the targeted phonemes for the purpose of making them more easily audible and facilitating focusing attention in those specific sounds (15).

**Cloze procedure with phonemic cues.** With this strategy, the clinician prompts the child’s communicative turn by supplying part of the utterance and letting the child fill the rest (22). If necessary, the clinician can provide the selected sound of the target word (9). For example, when reading a storybook, the clinician can say: “Yes, she was hungry, and she found three bowls of ____(soup) on the t t ____(table)”. The idea is that sharing the responsibility of telling the story and letting fill in the blank space, the children can focus on the specific words and the targeted sounds. Also, using phonemic cues by providing the initial sound or syllable the child can direct his/her attention to the targeted sound and the distinctive features of it.

**Phonetic changes.** The speech pathologist indicates that the message would be more easily interpreted with a modification in speech production (9). For example, when the child says “the _en (the pen)” reading a storybook in which there are different objects, the clinician says: “the pen”. Remember to put your lips together and make an explosion: p pen”. With this strategy, the information would directly contribute to refining the phonemic distinction being articulated.

**Think aloud in phonemic awareness.** Originally, this is a metacognitive strategy where the speech pathologist verbalizes thoughts while reading a selection, thus modeling the process of comprehension (23). This strategy enables to demonstrate the patient how to select an appropriate articulation process at a specific point in a particular communicative message. The clinician verbalizes specific think-aloud about different levels of language organization including information about the sounds of speech. For example, before reading a storybook, the clinician says: “Let us think which sounds we will be focusing on. We have to consider that some sounds are short and explosive like /k/, /p/, /t/. I will write these letters for reminding us to use them while we refer to the ideas and words of the book. Besides, we have other sounds that are long and continuous: /s/ or /f/. I will write these letters in this other paper. While looking at the storybook, the clinician is focusing on the target sounds and explaining the characteristics of each phoneme and how changing the sound can change the characteristics of the word increasing intelligibility. Also, the clinician gives specific instructions about how to produce sounds correctly (15).

It is important to emphasize, that strategies, which include modeling and modeling with stress, do not involve direct instruction, but they establish an appropriate context for focusing on the sounds of speech while receiving the correct model of articulation of the targeted sounds. In contrast, the rest of the strategies provide information for managing the sounds of speech. For this reason, for the purpose of this paper, they will be considered as strategies involving direct instruction.

As stated before, in this study, for the control group, the SLP used all the strategies in each case indistinctively. In contrast, in the experimental group the SLP applied the same strategies depending on the level of articulation (Table 1).

## RESULTS

The age of the patients from both groups ranged from 3 years to 6;8 years. The median age was 4;10 years.

Two independent examiners revised the videotaped phoniatric clinical evaluations for classifying patients according to the clinical scale for the severity of CA. They agreed in 95% of the cases. Whenever there was disagreement, each case was discussed until reaching a consensus.

At the onset of the speech therapy period, all patients included in both groups demonstrated CA. Both groups were similar at the beginning of the study and had similar severity levels of CA. A Mann Whitney test demonstrated a non-significant difference between groups at the onset of the summer camp (z sub t=1.89. *P*>0.05).

A student-t test demonstrated a non-significant difference between ages in both groups. Mean age in the control group was 60.27 mo. SD = 13.29. Mean age in the experimental group was 59.78 mo. SD=11.49. t=0.187, *P*=0.852 (Table 2 and Table 3).

**Table 2:** Level of articulation at the onset, at the end of the study, and levels of advance

Patient	Age	Level of articulation at the onset of the Camp	Level of articulation at the end of the Camp	Levels of advance

1	3;2	2	3	1
2	3;3	1	3	2
3	3;4	2	3	1
4	3;6	3	4	1
5	3;6	1	3	2
6	3;6	3	4	1
7	3;7	0	2	2
8	3;7	1	2	1
9	3;10	2	3	1
10	4;3	0	1	1
11	4;4	0	1	1
12	4;4	1	2	1
13	4;4	1	3	2
14	4;5	0	0	0
15	4;8	1	3	2
16	4;9	2	3	1
17	4;10	1	2	1
18	4;10	0	2	2
19	4;11	3	4	1
20	5;0	2	3	1
21	5;0	0	1	1
22	5;1	2	3	1
23	5;1	2	3	1
24	5;1	1	3	1
25	5;2	2	3	1
26	5;3	0	1	1
27	5;3	2	3	1
28	5;5	1	3	1
29	5;5	2	3	1
30	5;6	0	0	0
31	5;7	2	3	1
32	5;9	3	4	1
33	5;9	2	3	1
34	5:10	0	1	1
35	5:10	1	2	1
36	5:11	1	3	2
37	6;0	2	3	1
38	6;0	0	1	1
39	6;0	2	4	2
40	6;1	2	4	2
41	6;1	1	3	2
42	6;2	1	3	2
43	6;2	0	1	1
44	6;4	0	1	1
45	6;4	1	3	2

Indistinct use of strategies in the control group.

**Table 3:** Level of articulation at the onset, at the end of the study, and levels of advance

Patient	Age	Level of articulation at the onset of the Camp	Level of articulation at the end of the Camp	Levels of advance

1	3	1	3	2
2	3	1	3	2
3	3;1	2	4	2
4	3;2	3	4	1
5	3;4	2	4	2
6	3;4	1	3	2
7	3;5	0	3	3
8	3;7	2	4	2
9	3;10	3	4	1
10	4	0	2	2
11	4;1	2	4	2
12	4;4	1	3	2
13	4;4	0	3	3
14	4;6	0	4	4
15	4;6	1	3	2
16	4;8	1	3	2
17	4;9	1	3	2
18	4;11	1	3	2
19	4;11	2	4	2
20	4;11	0	3	3
21	5;1	0	3	3
22	5;2	0	3	3
23	5;3	2	4	2
24	5;3	0	2	2
25	5;3	1	3	2
26	5;4	0	3	3
27	5;4	0	3	3
28	5;6	2	4	2
29	5;7	0	2	2
30	5;7	2	4	2
31	5;8	0	2	2
32	5;9	1	3	2
33	5;9	1	4	3
34	5;9	0	3	3
35	5:10	1	4	3
36	5:10	1	4	3
37	5:11	3	4	1
38	6;0	2	4	2
39	6;1	1	3	2
40	6;3	0	4	4
41	6;5	2	3	1
42	6;7	2	4	2
43	6;7	2	4	2
44	6;8	1	3	2
45	6;8	1	4	3

Use of strategies according to the level of articulation in the experimental group.

When comparing levels of articulation at the onset and at the end of the study in each group, a Wilcoxon test showed a significant difference between levels in articulation in the control group, indicating an improvement in articulation during the summer camp (z sub w=5.77. *P*<0.0001) (Table 2).

Similarly, the experimental group showed an advance in articulation in all patients during the summer camp. A Wilcoxon test also showed a significant difference when comparing articulation levels at the onset and at the end of the study, (z sub w=6.06. *P*<0.0001) (Table 3).

**Figure 1:**
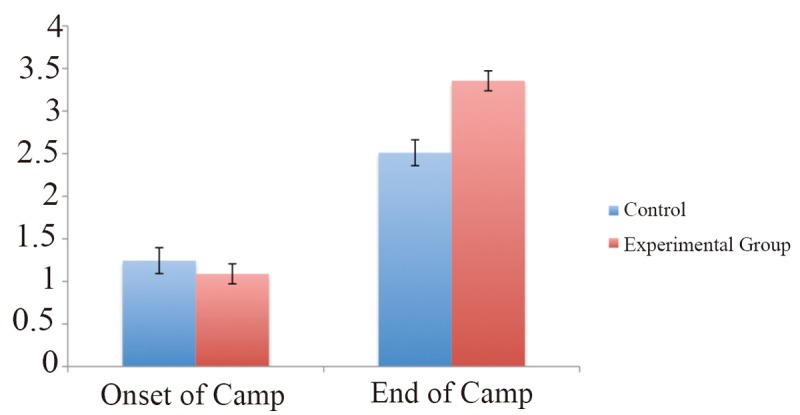
Mean level of articulation at the onset and at the end of the camp for each group. Children in the experimental group improved significantly more than in the control group.

Finally, when comparing the levels of advance in both groups, a Mann Whitney test showed a significant difference that indicates a greater advance in the experimental group, (z sub t=5.845. *P*<0.0001) (Figure 1).

## DISCUSSION

Several strategies have been described for scaffolding language and/or articulation. In a previous report, (15), found that some PCP showed a better response when some strategies were used during intervention. The purpose of this paper was to study and compare two different ways to use scaffolding strategies during speech therapy with PCP who were present with CA. It was hypothesized that considering the level of severity of articulation impairment would be useful for deciding which strategy would be more effective during intervention. The results of this study seemed to support this statement.

Phonological acquisition is gradual (24). It is a process. For assessment and intervention, it is important to understand the system of each child, and to recognize in which step of the process is at the moment. For this purpose, a clinical scale of severity of CA in PCP has been used in this study (19). The scale allows determining the moment of the process for correcting CA for each patient. That is, the degree of severity of CA in each child.

Considering the characteristics of the phonologic system of each child can be useful for facilitating the selection of some strategies which may be more effective than others in that particular case. Moreover, if the dynamics involved in each strategy are analyzed, some strategies appear to be more appropriate for different stages in the natural process of correcting a compensatory articulatory pattern in PCP.

The results of this study suggest that when speech strategies are used according to the severity of the compensatory articulatory pattern (which is classified depending on the frequency of its appearance during speech and the response to correction prompts), it seems easier for the child to follow the speech pathologist lead in order to correct the pattern.

Strategies that include direct instruction, including phonetic change, cloze procedure with phonemic cues, and think aloud in phonemic awareness, seem more appropriate for improving articulation in patients who show higher levels of severity of CA. In other words, patients who are able to produce a correct articulatory pattern only on isolated phonemes or short words, with consistent prompting are more likely to show an effective response to direct instruction on placement and manner of articulation. A possible explanation is that these patients show a limited level of phonologic awareness.

In contrast, strategies, which use modeling and modeling with stress usually, seem to be effective in patients with lower degrees of severity of CA. These patients appear to be more aware of the characteristics of the speech sounds. Thus, they seem to be more confident for producing these sounds during a more structured and complex discourse (15).

It should be pointed out that the significant relationship between the effectiveness of specific strategies with the severity of CA, seemed to be independent of the age of the patients. In a previous study, an effective correction of an abnormal articulatory pattern was not significantly correlated with the age of the patients (15).

In our center, most of the patients come from families with extremely low educational level and severe social and economical limitations. In this population, our group has previously reported that speech pathology treatment is more effectively provided when articulation is addressed within a linguistic context, as compared with a fragmented approach. (3). It is necessary to consider that besides the expected speech disorders in patients with cleft palate, there are reports describing that a significant percentage of these patients can also show language impairment (17). This increased prevalence of language impairment in patients with palatal clefts is more likely multifactorial, but the educational level of the parents and the social environment, have been reported as some of the causal factors (25). Improving speech intelligibility is extremely relevant for the child’s social and integral development and the final outcome result.

Although, the reduced and homogenized number of patients included in this study, precludes obtaining definite results, the conclusions of this paper seem useful and promising. It will be necessary to study larger numbers of patients in different situations, as well as include more strategies in future studies.

It can be concluded that selecting the speech pathology treatment strategies for each case, according with its specific characteristics, including the severity of the articulation disorder, as assessed by its use in conversational speech and the response to prompting, can become a valuable procedure for improving outcome during the speech intervention.
